# Racial/ethnic disparities in gastric cancer: A 15‐year population‐based analysis

**DOI:** 10.1002/cam4.4997

**Published:** 2022-07-03

**Authors:** Maria Gonzalez‐Pons, Carlos R. Torres‐Cintrón, Marievelisse Soto‐Salgado, Yimari Vargas‐Ramos, Luis Perez‐Portocarrero, Douglas R. Morgan, Marcia Cruz‐Correa

**Affiliations:** ^1^ Division of Cancer Biology University of Puerto Rico Comprehensive Cancer Center San Juan Puerto Rico USA; ^2^ Analysis and Epidemiology Unit Coordinator / Biostatistician, Puerto Rico Central Cancer Registry, Division of Cancer Control and Population Sciences University of Puerto Rico Comprehensive Cancer Center San Juan Puerto Rico USA; ^3^ Division of Cancer Control and Population Sciences University of Puerto Rico Comprehensive Cancer Center San Juan Puerto Rico USA; ^4^ Ponce Health Sciences University School of Medicine Ponce Puerto Rico USA; ^5^ Director of Division of Gastroenterology and Hepatology University of Alabama, Birmingham Birmingham Alabama USA; ^6^ University of Puerto Rico Comprehensive Cancer Center San Juan Puerto Rico USA; ^7^ Department of Medicine and Biochemistry University of Puerto Medical Sciences Campus San Juan Puerto Rico USA

**Keywords:** ethnicity, gastric cancer, health disparities, hispanics, race

## Abstract

Background and aims: Disparities in gastric cancer incidence and mortality have been reported among ethnic/racial groups. While gastric cancer is not common in the U.S., it is among the top 10 causes of cancer‐related death among Hispanics living in Puerto Rico (PRH). This study compared gastric cancer incidence rates during a 15‐year period (2002–2006, 2007–2011, and 2012–2016) between PRH and racial/ethnic groups in the mainland U.S., including Non‐Hispanic Whites (NHW), Non‐Hispanics Blacks (NHB), Hispanics (USH), and Non‐Hispanic Asian or Pacific Islanders (NHAPI).

**Methods:**

Primary gastric cancer cases (ICD‐O‐3 codes C16.0 to C16.9) from the Puerto Rico Central Cancer Registry and SEER diagnosed from January 1, 2002 to December 31, 2016 were included in the analysis. The Joinpoint Regression Program and standardized rate ratios were used to estimate Annual Percent Changes (APC) and differences in gastric cancer incidence among racial/ethnic groups, respectively.

**Results:**

Our analysis included 83,369 gastric cancer cases (PRH *n* = 4202; NHW *n* = 43,164; NHB *n* = 10,414; NHAPI *n* = 11,548; USH *n* = 14,041). USH had the highest number of cases among individuals <50 years, whereas NHW and PRH had the highest percentage among individuals ≥50 years. PRH and USH were the only groups with increasing APCs among individuals <50 years.

**Conclusions:**

Gastric cancer continues to be a common cancer among PRH, despite the overall decrease in incidence among other racial/ethnic groups. Studies evaluating the gastric cancer risk factors among high‐risk groups are necessary to establish health policy and modify gastric cancer screening algorithms among Hispanics.

## Graphical abstract

Among Hispanics, gastric tumors continue to be commonly diagnosed; only 32% of individuals diagnosed with this malignancy survive more than 5 years. An increase in gastric cancer among individuals younger than 50 years and diagnosis at regional (more advanced) stages was observed when comparing Hispanics living in Puerto Rico to other racial/ethnic groups on the U.S. mainland. Our findings underscore the importance of evaluating gastric cancer risk factors among diverse, high‐risk groups, such as Hispanics, in order to develop tailored prevention and risk stratification strategies.

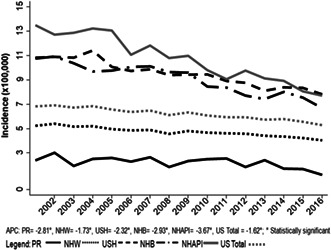

## 1 INTRODUCTION

Globally, gastric cancer is the 5th most frequently diagnosed malignancy^1^; more than one million individuals are diagnosed with gastric cancer each year.^1^ Patients with gastric cancer have a dismal prognosis; only 32% of individuals diagnosed with this malignancy survive >5 years.^2^ Although gastric cancer is not commonly diagnosed in the U.S., in 2022 approximately 26,380 individuals will be diagnosed and 11,090 patients will succumb to this malignancy.^3^ Gastric cancer is the 15th most commonly diagnosed malignancy in Puerto Rico, and the 6th and 8th leading cause of cancer‐related death among men and women, respectively.^4^

Infection with *Helicobacter pylori*, sex, age, and race/ethnicity, among others, are all risk factors associated with the development of gastric tumors.^2^ *Helicobacter pylori*, a Group 1 carcinogen, is a predominant predisposing factor for gastric carcinogenesis.^5^ The median age of diagnosis is 68 years in the U.S.; however, an increase in gastric cancer incidence rates has been reported among individuals younger than 50 years.^6^, ^7^ Differences in gastric cancer incidence have been reported according to sex; men have been reported to be twice as likely to develop gastric tumors than women.^1^, ^8^ Despite living in the same regions, the overall incidence of gastric cancer among racial/ethnic groups has been significantly variable.^2^ In the U.S., non‐Hispanic Blacks (NHB), Hispanics (USH), and non‐Hispanic Asian or Pacific Islander (NHAPI) are more commonly diagnosed with gastric tumors compared to Non‐Hispanic Whites (NHW).^9^ Moreover, significant disparities in gastric cancer survival have also been reported according to race/ethnicity, with Asians having higher survival rates compared to NHW, NHB, and Hispanics.^10^

Data from the 2019 U.S. CENSUS showed that approximately 36% of the total U.S. population is comprised of individuals with diverse ethnic/racial backgrounds from countries with high gastric cancer incidence. Puerto Ricans are currently the 2nd largest Hispanic subpopulation, with approximately 5.8 million individuals residing on the mainland and another 3.1 million living on the island.^11^ Most epidemiological studies in the U.S. aggregate Hispanic subpopulations under “Hispanics,” which may conceal significant variability in gastric cancer incidence and mortality in Hispanic subgroups according to their country of origin. In the present study, we report the overall trends of gastric cancer incidence rates during 2002–2016, and the relative risk of incidence by demographic and clinical characteristics by comparing Hispanics living in Puerto Rico (PRH) with racial/ethnic groups from the mainland U.S. We examined a Hispanic subpopulation with a disproportionate gastric cancer burden and provide evidence to guide future prevention strategies and health policies to ultimately reduce gastric cancer mortality in Puerto Rico.

## 2 MATERIALS AND METHODS

### 2.1 Data sources

Gastric cancer incidence rates from 2002 to 2016 for Puerto Rico were provided by the Puerto Rico Central Cancer Registry (PRCCR). The PRCCR collects demographic and clinical information, including pathology (anatomic site, tumor histology, stage at diagnosis [localized, regional or distant]) and treatment. The PRCCR obtains the cancer‐related digital death files from the Demographic Registry of the Puerto Rico Department of Health. The North American Association of Central Cancer Registries (NAACCR) guidelines are used by the PRCCR for coding, data gathering, and reporting. The 3rd edition of the International Classification of Diseases for Oncology (ICD‐03) was used to report all cancer cases diagnosed throughout the study period (2002–2016). Only primary cases with a diagnostic and histologic confirmation of gastric cancer (ICD‐03 codes: C16.0–C16.9) from January 1, 2002 to December 31, 2016 were included in our study.

Gastric cancer incidence rates from 2002 to 2016 for the racial/ethnic groups included in our study (NHB, USH, NHW, and NHAPI) were obtained from the Surveillance, Epidemiology, and End Results (SEER) Program. To detect individuals with Hispanic ethnicity, SEER uses a combination of medical record review and matching surnames against a list of Hispanic surnames. The term Hispanic, which we employ throughout our analyses, does not consider racial distinctions among the USH population.

### 2.2 Age‐standardized rates

Using the 2000 U.S. standard population, the gastric cancer burden was calculated in all racial/ethnic groups using the direct method to calculate the age‐standardized rates (ASR) of gastric cancer incidence from 2012 to 2016 (per 100,000 persons).^12^ To determine the relative differences in gastric cancer incidence among groups, the Poisson regression models were used to estimate age‐specific relative risks (RR) with a 95% CI.^13^ The overall ASRs of gastric cancer incidence for each racial/ethnic group were then calculated with 95% CI. The standardized rate ratio (SRR), a ratio of two standardized rates between different groups, was estimated with 95% CI to assess differences in gastric cancer incidence rates between PRH, NHB, USH, NHW, and NHAPI.^14^ Statistical analysis was performed using STATA v17.0.

### 2.3 Annual percent change

We used the Joinpoint Regression Program (Version 4.9.0.0) to calculate the annual percent change (APC) and 95% CI.^15^ The methodology used by this program employed several permutation tests and identified the best‐fit line through the 15‐year period of annual age‐standardized incidence rates to determine the number of significant joinpoints.^16^

## 3 RESULTS

### 3.1 Gastric cancer incidence by racial/ethnic group

**FIGURE 1:**
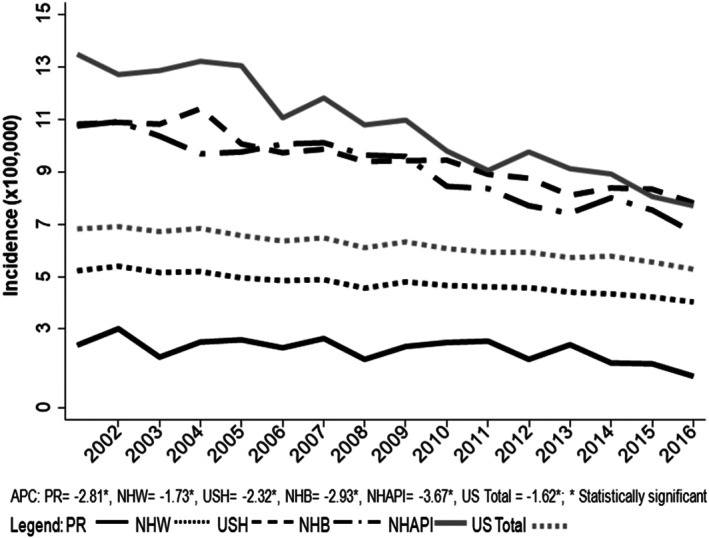
Trends for ASRs of gastric cancer incidence (per 100,000) by ethnic/racial group from 2002 to 2016.

During 2002–2016, a total of 83,369 cases of gastric cancer were identified. Overall, incidence rates continuously declined in all racial‐ethnic groups during this period (Figure 1). Demographic and clinical characteristics for incident gastric cancer by race/ethnicity are shown in Table 1. Among the racial/ethnic groups studied, USH were the racial/ethnic group with the lowest median age, the highest percentage of cases diagnosed in individuals <50 years, and the highest percentage of cases diagnosed at distant stages. PRH had an increase in the percentage of cases diagnosed at distant stages in each 5‐year period (data not shown), and the highest percentage of cases at regional stages.

**TABLE 1:** Demographic and clinical characteristics for incident gastric cancer by race/ethnicity, 2002–2016

Characteristic	PRH	NHW	NHB	NHAPI	USH
	*n* = 4202 (%)	*n* = 43,164 (%)	*n* = 10,414 (%)	*n* = 11,548 (%)	*n* = 14,041 (%)
Age group					
<50	276 (6.6)	2897 (6.7)	1167 (11.2)	1280 (11.1)	2856 (20.3)
50+	3926 (93.4)	40,267 (93.3)	9247 (88.8)	10,268 (88.9)	11,185 (79.7)
IQ50 (IQ25, IQ75)	72 (63, 81)	71 (61, 80)	68 (57, 78)	59 (71, 80)	52 (65, 76)
Sex					
Male	2527 (60.1)	28,879 (66.9)	6184 (59.3)	6638 (57.5)	8207 (58.5)
Female	1675 (39.9)	14,285 (33.1)	4230 (40.6)	4910 (42.5)	5834 (41.5)
Stage at diagnosis					
Localized	1343 (32.0)	10,132 (23.5)	2365 (22.7)	2945 (25.5)	2648 (18.9)
Regional	1414 (33.7)	12,214 (28.3)	3016 (28.9)	3716 (32.2)	4106 (29.2)
Distant	811 (19.3)	16,527 (38.3)	3964 (38.1)	3791 (32.8)	6018 (42.9)
Unknown	634 (15.0)	4291 (9.9)	1069 (10.3)	1096 (9.5)	1269 (9.0)

*Note*: Abbreviations: NHAPI, Non‐Hispanic Asian or Pacific Islanders; NHB, Non‐Hispanic‐Blacks; NHW, Non‐Hispanic Whites; PRH, Puerto Rico; USH, U.S. Hispanics.

The overall ASR of gastric cancer incidence per 100,000 by racial/ethnic group from 2012–2016 are shown in Table 2. NHAPI had the highest age‐standardized incidence rate overall, followed by USH, NHB, PRH, and NHW. The same trend was observed when analyzing age‐standardized incidence according to sex, with the exception that NHB men had a slightly higher incidence than USH. Among all of the racial/ethnic groups studied, men had higher standardized incidence rates than women. PRH had a lower risk of gastric cancer incidence overall compared to USH (SRR = 0.69; 95% CI = 0.66–0.72), NHB (SRR = 0.76; 95% CI = 0.73–0.80), and NHAPI (SRR = 0.66; 95% CI = 0.63–0.69). When comparing PRH and NHW, PRH had a significantly higher risk of overall gastric cancer incidence (SRR = 1.31; 95% CI = 1.26–1.37). A higher risk of cancer incidence was observed among PRH men (SRR = 1.19; 95% CI = 1.13–1.26) and women (SRR = 1.63; 95% CI = 1.52–1.74) compared to NHW men and women, respectively.

**TABLE 2:** Age‐standardized incidence rates (per 100,000) for gastric cancer from 2012 to 2016

	Age‐standardized rates^a^						RR (95% CI)^b^				
	PR	NHW	USH	NHB	NHAPI	U.S. overall	PR vs. NHW	PR vs. USH	PR vs. NHB	PR vs. NHAPI	PR vs. U.S. overall
Overall	5.68	4.33	8.25	7.47	8.66	5.65	1.31 (1.26–1.37)	0.69 (0.66–0.72)	0.76 (0.73–0.80)	0.66 (0.63–0.69)	1.00 (0.96–1.05)
Male	7.82	6.55	10.68	10.78	11.58	8.02	1.19 (1.13–1.26)	0.73 (0.69–0.78)	0.73 (0.68–0.77)	0.68 (0.64–0.72)	0.97 (0.93–1.03)
Female	4.03	2.48	6.38	5.17	6.44	3.74	1.63 (1.52–1.74)	0.63 (0.59–0.68)	0.78 (0.72–0.84)	0.63 (0.58–0.67)	1.08 (1.01–1.15)

*Note*: Abbreviations: NHAPI, non‐Hispanic Asian or Pacific Islanders; NHB, non‐Hispanic black; NHW, non‐Hispanic white; PR, Puerto Ricans; U.S. overall, All U.S. racial/ethnic groups; USH, U.S. Hispanics.

^a^ Age‐standardized rates using the U.S. 2000 standard population.

^b^ SSR indicated standardized rate ratio with a 95% confidence interval.

### 3.2 Annual percent changes by racial/ethnic group

The temporal trend of overall gastric cancer incidence is shown in Table 3. PRH had a marked increase in incidence rates among individuals <50 years of age (APC = 4.2; 95% Cl = −2.0‐10.9) and gastric cancer diagnosis at regional stages (APC = 1.6; 95% Cl = 0.0–3.2) compared to NHB, USH, NHW, and NHAPI. A slight increase in the incidence rates of individuals <50 years of age was also observed in USH. Only one increasing joinpoint was observed among USH with regional gastric cancer during 2014–2016; however, this increase was not significant (data not shown).

**TABLE 3:** Annual percent change (APC) in gastric cancer incidence by race/ethnic group 2002–2016

	PRH	NHW	NHB	NHAPI	USH
	APC (CI)	APC (CI)	APC (CI)	APC (CI)	APC (CI)
Overall	−2.8^a^ (−4.8 to −0.7)	−1.6^a^ (−1.9 to −1.4)	−2.9^a^ (−3.5 to −2.4)	−3.7^a^ (−4.2 to −3.1)	−2.3^a^ (−2.7 to −1.9)
Age group					
<50	**4.2** (−2.0 to 10.9)	−0.5 (−1.4 to 0.4)	−2.1^a^ (−3.0 to −1.2)	−2.6^a^ (−4.0 to −1.3)	**0.4** (−0.3 to 1.1)
50+	−3.2^a^ (−5.3 to −1.1)	−1.7^a^ (−2.0 to −1.5)	−3.0^a^ (−3.6 to −2.4)	−3.8^a^ (−4.4 to −3.2)	−2.7^a^ (−3.1 to −2.3)
Sex					
Male	−2.6 (−5.3 to 0.3)	−1.7^a^ (−2.1 to −1.4)	−3.2^a^ (−3.9 to −2.4)	−3.7^a^ (−4.3 to −3.1)	−2.5^a^ (−3.1 to −1.9)
Female	−3.5^a^ (−5.8 to −1.1)	−1.9^a^ (−2.3 to −1.5)	−2.8^a^ (−3.7 to −1.9)	−3.7^a^ (−4.4 to −3.0)	−2.2^a^ (−2.8 to −1.6)
Stage at diagnosis					
Localized	−5.1^a^ (−6.3 to −3.9)	−0.6^a^ (−1.2 to 0.0)	−3.2^a^ (−4.2 to −2.1)	−3.9^a^ (−5.1 to −2.6)	−2.2^a^ (−3.4 to −1.0)
Regional	**1.6** ^a^ (0.0 to 3.2)	−3.3^a^ (−3.8 to −2.7)	−4.3^a^ (−5.4 to −3.1)	−5.3^a^ (−6.1 to −4.5)	−3.9^a^ (−4.6 to −3.2)
Distant	−6.2^a^ (−8.1 to −4.3)	−0.3 (−0.8 to 0.1)	−1.3^a^ (−2.2 to −0.3)	−2.3^a^ (−3.2 to −1.5)	−1.0^a^ (−1.7 to −0.3)

*Note*: The 95% confidence interval is given in *parentheses*.

^a^ Annual percent change (APC) is significantly different from zero at the alpha = 0.05 level.

## 4 DISCUSSION

Even though gastric cancer is not common among NHW, racial/ethnic disparities in the U.S. persist, with NHAPI having the highest gastric cancer incidence rates, followed by USH and NHB.^17^ Non‐White individuals have been reported to be approximately 1.5–2.0 times more likely than NHWs to be diagnosed with gastric cancer and succumb to this malignancy.^18^, ^19^, ^20^, ^21^ However, aggregating heterogeneous populations in epidemiological studies, such as Hispanics, conceal the significant variability that may exist in gastric cancer incidence and mortality among Hispanic subgroups according to their country of origin. Differences in the incidence of gastric cancer have been reported among Hispanic subgroups according to their country of origin.^22^, ^23^ According to the 2019 U.S. Census, individuals with diverse ethnic/racial origins account for 36% of the total population; 18.5% are Hispanics. Puerto Ricans are the second largest subpopulation of Hispanics in the U.S. and account for approximately 10% of Hispanics, which underscores the importance of characterizing gastric cancer incidence trends in this Hispanic subpopulation with a disproportionate cancer burden.^3^ In the present study, we describe the gastric cancer incidence trends among Hispanics living in Puerto Rico (PRH) compared to other racial/ethnic groups in the mainland U.S. during a 15‐year period.

An overall decreasing gastric cancer incidence trend during 2002–2016 in all of the racial/ethnic groups studied was observed. This is not surprising as a reduction in overall gastric cancer incidence rates has been reported in the last 50 years.^2^, ^24^, ^25^ The decreasing trends in gastric cancer incidence rates have been attributed to improvements in sanitation, clean water, food preservation, and *Helicobacter pylori* screening and treatment.^18^ The majority of gastric cancer cases observed during our study period (2002–2016) were among males, individuals older than 50 years, and were mostly diagnosed at distant, advanced stages. In the U.S., men are twice as likely to be diagnosed with gastric cancer than women, and the majority of patients diagnosed with gastric tumors are between the ages of 65 and 74 years of age.^2^, ^17^, ^26^ Similar to what has been previously published, USH had the highest percentage of cases diagnosed among the <50 years age group compared to the racial/ethnic groups studied.^27^ Differences in lifestyle, area‐based socioeconomic position, and environmental exposures, such as earlier exposure to *H. pylori*, may in part explain the differences observed. Differences in demographic and immigration patterns among Hispanic populations, including *H. pylori* prevalence and virulence in their country of origin, could be contributing to the observed trend among USH. When comparing stages at diagnosis, the majority of racial/ethnic groups examined were diagnosed at distant stages, with the exception of PRH. A possible explanation for the lower number of gastric tumors diagnosed at advanced stages among PRH could be that amidst the high percentage of cases without tumor staging information (unknown stages), there could be a significant number of unclassified tumors at advanced, distant stages. As the number of tumors diagnosed without staging information decreased in each 5‐year period, we observed a concomitant increase in the percentage of cases diagnosed at distant stages, thereby supporting our notion. Another aspect that may be contributing to the observed trends may be increased endoscopic surveillance by local gastroenterologists as there have been national/international professional society guidelines recommending endoscopic screening for those at higher risk of developing gastric cancer, such as ethnic background.^28^ Furthermore, an evaluation of detection methods and reports of the PRH population are needed, such as elucidating the why there is a high number of cases diagnosed as unknown stage.

The highest overall age‐standardized gastric cancer incidence rates were observed among NHB, USH, and NHAPI during 2012–2016. Although gastric cancer is uncommon in the U.S., a significantly higher incidence and mortality rates among these three racial/ethnic subgroups has been previously described compared to NHW.^29^ These disparities in gastric cancer incidence can partly be explained by the fact that in the U.S., approximately 29% of foreign‐born individuals of diverse ethnic backgrounds come from countries with high gastric cancer incidence rates.^4^ The majority of all new gastric cancer cases worldwide, approximate 50% of all cases are among individuals in Asian‐Pacific countries, followed by followed by Eastern European, and Central and Latin American countries.^4^ Differences in the prevalence of *H. pylori* infections, dietary patterns, and socioeconomic status have been associated with the geographical variation in gastric cancer incidence.^30^ The prevalence of *H. pylori* infections varies dramatically by geographic region with high prevalence rates in Asia (54.7%), Latin America (63.4%), and Africa (79.1%).^31^ The prevalence of *H. pylori* in the U.S. was approximately 31%, with variable prevalence across racial/ethnic groups: 21% in Whites, 52% in African Americans, and 64% in Mexican Americans^32^. Asian immigrants have also been reported to have high *H. pylori* seroprevalence rates (70%).^33^ Among PRH, seroprevalence rates (33%) were comparable to the overall *H. pylori* seroprevalence rates reported in the continental U.S. (30.7%).^32^, ^34^ Lower socioeconomic status, which has been reported as a risk factor for both *H. pylori* and gastric cancer, may also in part explain the observed racial/ethnic disparities in gastric cancer incidence.^21^, ^35^, ^36^ NHB and USH have a very low socioeconomic index, with 20.8% and 17.6% poverty rates, respectively, compared to 10.1% for Asians and 8.1% for NHW^37^. PRH also have a very low socioeconomic index, with an average household median income of $20,539, which is nearly 50% lower than NHB, which are the racial/ethic group with the lowest median income in the mainland U.S.^37^, ^38^ However, *H. pylori* seroprevalence rates were markedly lower among PRH than in NHB, USH, and NHAPI,^34^ which may in part explain why PRH have lower gastric cancer incidence rates, despite having a very low socioeconomic index.

Although PRH have a comparable gastric cancer risk compared to the population living on the U.S. mainland overall, a significantly higher risk was observed when compared to NHW, with the highest risk observed among women. The lower socioeconomic index among PRH compared to NHW may in part explain why this Hispanic subpopulation has a higher risk of developing gastric tumors. A higher prevalence of autoimmune diseases leading to autoimmune atrophic gastritis (a known etiological factor for gastric intestinal metaplasia, a precancerous lesion that most commonly affects females) may in part explain the higher gastric cancer risk among PRH women compared to NHW women. PRH women have a higher prevalence of autoimmune diseases, such as diabetes and systemic lupus erythematosus, compared to NHW^39^, ^40^; hence, autoimmune gastritis among PRH women may be an important etiological factor that needs further evaluation. Women are more commonly diagnosed with autoimmune gastritis than men (3:1 ratio), and close to 10% of patients with autoimmune gastritis develop gastric cancer.^41^, ^42^ The fact that gastric cancer continues to be one of the top 10 causes of cancer‐related death in Puerto Rico underscores the need for future studies to identify additional risk factors that may contribute to the disparities observed.

Decreasing gastric cancer incidence trends were observed overall among all the racial/ethnic groups studied; however, we observed a marked increase in gastric cancer among PRH <50 years old and gastric cancer diagnosis at regional stages. USH was the only other racial/ethnic group with a slight increase in gastric cancer among young individuals. Possible factors that could contribute to the higher trends in the incidence of gastric cancer among Hispanics could be that this population could have a higher prevalence of autoimmune gastritis‐driven tumors, hereditary gastric cancer, and/or epigenetic changes due to environmental exposures. Although familial aggregation is observed in approximately 10% of gastric cancers, a higher prevalence of hereditary gastric cancer among PRH and USH may partly explain the increasing trend in early‐onset gastric cancer incidence.^43^ Changes in epigenetic drivers of gastric carcinogenesis, as a result of environmental exposures among Hispanics, including dietary and lifestyle factors (e.g., consumption of saturated fat, smoking, etc.) and consequent microbiome dysbiosis, may also contribute to the observed increase in early‐onset trends among in this racial/ethnic subgroup.^44^, ^45^, ^46^ Another possible explanation could be that Hispanics have an earlier presentation and higher prevalence of autoimmune disease‐driven gastric cancer. For example, the prevalence of systemic lupus erythematosus is very high in Puerto Rico, and a higher percentage of Hispanics have been reported to be diagnosed with systemic lupus erythematosus before the age of 50 compared to NHW, NHB, and NHAPI.^40^, ^47^ The observed increase in gastric cancer diagnosis at regional stages among PRH may be due to increased awareness of the disease and gastric screening education among GI practitioners. However, due to the high percentage of cases with missing data regarding the stage of diagnosis, we must exercise caution in interpreting incidence trends according to stage at diagnosis in PRH. Additional research is warranted to examine risk factors and possible gene–environment interactions that contribute to these early‐onset gastric cancer disparities are needed to develop risk stratification and prevention strategies for Hispanic individuals at risk.

In sum, the present study showed that although overall gastric cancer incidence has been consistently decreasing, racial/ethnic disparities persisted during the 15‐year study period (2002–2016). The racial/ethnic disparities observed, especially the increasing incidence of early‐onset gastric cancer among Hispanics, warrant further investigation of the risk factors contributing to an earlier presentation of gastric tumors. Limitations in our study include a higher percentage of gastric tumors diagnosed at unknown stages among PRH and limited information regarding tumor location, which limited our ability to evaluate the impact of tumor stage at diagnosis and location (cardia vs. non‐cardia tumors) in our analyses. Nonetheless, our findings underscore the importance of examining gastric cancer trends, including examining gastric cancer survival, in heterogeneous populations such as Hispanics, which are crucial to inform tailored prevention and risk stratification strategies to reduce the gastric cancer burden in subpopulations at higher risk.

## AUTHOR CONTRIBUTIONS

Maria Gonzalez‐Pons: Conceptualization: Equal; Formal analysis: Equal; Methodology: Equal; Writing – original draft: Lead; Writing – review & editing: Lead. Carlos R. Torres‐Cintrón: Data curation: Lead; Formal analysis: Lead; Methodology: Lead; Writing – original draft: Supporting; Writing – review & editing: Supporting. Marievelisse Soto‐Salgado: Data curation: Supporting; Formal analysis: Equal; Methodology: Supporting; Writing – original draft: Supporting; Writing – review & editing: Supporting. Yimari Vargas‐Ramos: Data curation: Supporting; Formal analysis: Supporting; Writing – original draft: equal. Luis Perez‐Portocarrero: Formal analysis: Supporting; Writing – original draft: Supporting; Writing – review & editing: Supporting. Douglas Morgan: Conceptualization: Equal; Writing – original draft: Supporting; Writing – review & editing: Equal. Marcia Cruz‐Correa: Conceptualization: Lead; Resources: Lead; Writing – original draft: Equal; Writing – review & editing: Equal.

## FUNDING INFORMATION

This project was supported by the Puerto Rico Central Cancer Registry (CDC/National Program Cancer Registries Award Number U58 DP006318) and by a diversity supplement awarded to Marievelisse Soto‐Salgado tied to NCI Award Number CA096297.

## CONFLICT OF INTEREST

The authors do not have any conflict of interest to disclose.

## ETHICS APPROVAL

This study was approved by the University of Puerto Rico IRB (#A2210615).

## Data Availability

The data analyzed in this study is available through the Puerto Rico Central Cancer Registry and the Surveillance, Epidemiology, and End Results (SEER) Program.
